# The Protective Role of Prolyl Oligopeptidase (POP) Inhibition in Kidney Injury Induced by Renal Ischemia–Reperfusion

**DOI:** 10.3390/ijms222111886

**Published:** 2021-11-02

**Authors:** Giovanna Casili, Alessio Ardizzone, Rossella Basilotta, Marika Lanza, Alessia Filippone, Irene Paterniti, Emanuela Esposito, Michela Campolo

**Affiliations:** Department of Chemical, Biological, Pharmaceutical and Environmental Sciences, University of Messina, Viale Ferdinando Stagno d’Alcontres, 31-98166 Messina, Italy; gcasili@unime.it (G.C.); aleardizzone@unime.it (A.A.); rossella.basilotta@gmail.com (R.B.); mlanza@unime.it (M.L.); afilippone@unime.it (A.F.); ipaterniti@unime.it (I.P.); campolom@unime.it (M.C.)

**Keywords:** kidney ischemia reperfusion (KI/R), acute kidney injury (AKI), prolyl oligopeptidase (POP), inflammation, apoptosis, angiogenesis

## Abstract

Ischemia/reperfusion injury (IRI) is a complex pathophysiological process characterized by blood circulation disorder caused by various factors, such as traumatic shock, surgery, organ transplantation, and thrombus. Severe metabolic dysregulation and tissue structure destruction are observed upon restoration of blood flow to the ischemic tissue. The kidney is a highly perfused organ, sensitive to ischemia and reperfusion injury, and the incidence of renal IRI has high morbidity and mortality. Several studies showed that infiltration of inflammatory cells, apoptosis, and angiogenesis are important mechanisms involved in renal IRI. Despite advances in research, effective therapies for renal IRI are lacking. Recently it has been demonstrated the role of KYP2047, a selective inhibitor of prolyl oligopeptidase (POP), in the regulation of inflammation, apoptosis, and angiogenesis. Thus, this research focused on the role of POP in kidney ischemia/reperfusion (KI/R). An in vivo model of KI/R was performed and mice were subjected to KYP2047 treatment (intraperitoneal, 0.5, 1 and 5 mg/kg). Histological analysis, Masson’s trichrome and periodic acid shift (PAS) staining, immunohistochemical and Western blots analysis, real-time PCR (RT-PCR) and ELISA were performed on kidney samples. Moreover, serum creatinine and blood urea nitrogen (BUN) were quantified. POP-inhibition by KYP2047 treatment, only at the doses of 1 and 5 mg/kg, significantly reduced renal injury and collagen amount, regulated inflammation through canonical and non-canonical NF-κB pathway, and restored renal function. Moreover, KYP2047 modulated angiogenesis markers, such as TGF-β and VEGF, also slowing down apoptosis. Interestingly, treatment with KYP2047 modulated PP2A activity. Thus, these findings clarified the role of POP inhibition in AKI, also offering novel therapeutic target for renal injury after KI/R.

## 1. Introduction

Acute kidney injury (AKI) is a major medical problem [1], associated with high morbidity, mortality, and increased costs of treatment in both adult and pediatric population [2]. Renal ischemia/reperfusion (I/R) injury is a common cause of AKI; this injury initiates complex events within the kidney in renal injury and death of renal cells [3]. The inevitable injuries may occur after infarction, sepsis, and organ transplantation, and this phenomena exacerbate tissue damage by initiating an inflammatory cascade, including reactive oxygen species (ROS), cytokines, chemokines, and leukocytes activation [4]. The molecular mechanisms of AKI remain poorly understood and no effective therapeutic strategies to target AKI are available [5]. Therefore, novel therapeutic solutions need to be explored to improve the outcomes of AKI. In the kidney, IRI contributes to pathological conditions, called acute kidney injury (AKI), which is a clinical syndrome with rapid kidney dysfunction and high mortality rates [6]. The pathophysiology of KI/R is very complex, but some pathological pathways, such as activation inflammatory mediators, release of neutrophils, apoptosis, and angiogenesis are involved.

Inflammation as a common abnormality in kidney KI/R seems to link the various cell types, playing an important role in its pathophysiology [7]. Renal IRI triggers an inflammatory cascade that are involved in more renal damages, so inhibition of inflammatory responses is a therapeutic approach to protect renal tissue [8]. Pro-inflammatory cytokines and cytokines, such as interleukin 6 (IL-6) and TNF-α, play a major role in renal dysfunction of IRI [9]. 

Beyond inflammation, a number of pathologic processes contribute to AKI, including endothelial and epithelial cell death, intratubular obstruction, and changes in local microvascular blood. Particularly, during KI/R, the failure in renal function results in a stimulation of apoptosis, which significantly contributes to ischemic renal dysfunction [10].

The early stages of KI/R seem to be associated with an antiangiogenic response, whereas the hypoxia, which follows IR at later stages, may activate angiogenic factors, such as vascular endothelial growth factor (VEGF), and may be beneficial by stabilizing the microvasculature and favoring local blood supply [11]. On the other hand, reductions in microvasculature density may play a critical part in the progression of chronic kidney disease following initial recovery from IR injury [12]. 

A comprehensive analysis of serum peptidases activities in patients with chronic kidney disease (CKD) at different disease stages, noted as an alteration in peptidases/proteases activity in the renin–angiotensin system (RAS), is associated with the dysregulation of RAS axes and, consequently, to renal diseases [13]. Among the serum proteases associated with CKD, prolyl endo oligopeptidase (PREP or POP) plays a key role; it is found in all tissues, but it is localized only in specific cell types particularly in the brain, kidney, and liver [14]. Considering that inflammation is a focal point in AKI evolution [15] and knowing the direct involvement of POP in several inflammatory diseases [16], paying attention to POP-inhibitors as 4-phenyl-butanoyl-l-prolyl-2(S)-cyanopyrrolidine (KYP2047) could be the most potent strategy to counteract an inflammatory disease as renal ischemia. Based on these findings, the aim of this research was to evaluate the beneficial outcomes of KYP2047 treatment on POP-inhibition in AKI induced by an experimental mouse model of kidney ischemia/reperfusion.

## 2. Results

### 2.1. The Role of POP-Inhibition to Restore Histological Damage in AKI

**Figure 1:**
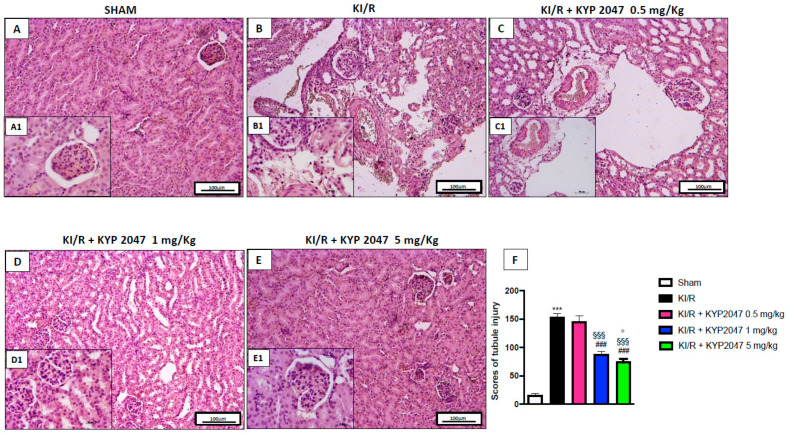
Role of KYP2047 treatment on histological damage induced by KI/R. H&E staining. Histopathologic examination of kidney samples in sham group (**A**,**A1**); severe histological damage with tubular alteration KI/R group (**B**,**B1**); treatment with KYP2047 0.5 mg/kg, 1 mg/kg, and 5 mg/kg (**C**,**C1**,**D**,**D1**,**E**,**E1**); tubular injury score (**F**). Magnification 10×, scale bar 100 μm (**A**–**E**) and 40×, scale bar 20 μm (**A1**–**E1**). Data represent the means of at least three independent experiments. One-way ANOVA followed by Bonferroni post-hoc. *** *p* < 0.001 versus Sham; ### *p* < 0.001 versus KI/R; §§§ *p* < 0.001 versus KI/R+ KYP2047 0.5 mg/kg; ° *p* < 0.05 versus KI/R+ KYP2047 1 mg/kg.

AKI represents a clinical syndrome with rapid renal dysfunction, histologically is characterized by major reductions in glomerular filtration rate, extensive tubular damage, tubular cell necrosis, glomerular injury, and signs of tubular obstruction with cell debris [17,18]. POP is ubiquitously present, revealing high activity in renal cortex, and the serum protease activity of POP was identified as closely associated with kidney function [13,19,20]. In this study, a significant histological alteration was observed in renal samples from KI/R (Figure 1(B,B1), see tubular injury score 1F) compared to control group (Figure 1(A,A1), see tubular injury score 1F). The POP-inhibition, mediated by treatment with KYP2047, significantly restored kidney dysfunction observed during the 6 h of reperfusion, at both doses of 1 and 5 mg/kg (Figure 1(D,D1,E,E1), see tubular injury score 1F). The treatment with KYP2047, at the lowest dose of 0.5 mg/kg, did not significantly improve the histological tubular alteration provoked by KI/R (Figure 1(C,C1), see tubular injury score 1F), and for this reason, it was decided to continue the analysis only with the higher doses that resulted in being protective.

### 2.2. The Effects of KYP2047 to Improve KI/R Dysfunction and Renal Markers

**Figure 2:**
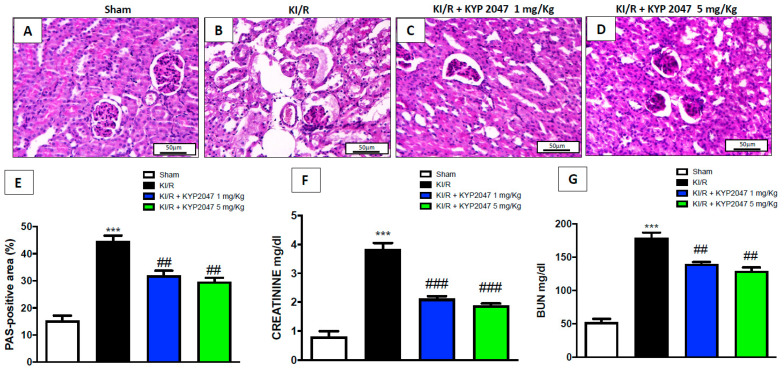
Role of KYP2047 treatment to improve KI/R dysfunction and renal markers. PAS staining evaluation of KI/R dysfunction in KI/R-injured group (**B**) compared to the control group (**A**); treatment with KYP2047 1 mg/kg and 5 mg/kg (**C**,**D**); % PAS-positive area (**E**). Magnification 20×, scale bar 50 μm (**A**–**D**). Serum evaluation of creatinine (Cr) and blood nitrogen urea (BUN) expressed as mg/dl (**F**,**G**). Data represent the means of at least three independent experiments. One-way ANOVA followed by Bonferroni post-hoc. *** *p* < 0.001 versus Sham; ### *p* < 0.001 and ## *p* < 0.01 versus KI/R.

KI/R provokes a wide loss of brush border, degeneration of tubular epithelial cells from the basement membrane, tubular cell necrosis. In this study, PAS staining was used to highlight renal injury based on tubular atrophy, blebbing tubular structures, and irregular tubular cytoplasm [21]; the results obtained, strengthening the histological analysis, described a significant tubular atrophy highlighted by the PAS-positive area in KI/R-injured mice (Figure 2B, see PAS-positive area Figure 2E) compared to control mice (Figure 2A, see PAS-positive area Figure 2E). The treatment with KYP2047, at both doses of 1 and 5 mg/kg, significantly lowered the percentage (%) of PAS-positive area (Figure 2C,D, see PAS-positive area Figure 2E). Despite the multifaceted nature of AKI, retrospective estimation of a single aspect of kidney function, using changes in serum markers remains the gold standard for phenotyping this complex disease [22]. Specifically, traditional markers of kidney function such as creatinine (Cr) and urea nitrogen must be evaluated in light of a possible altered balance [23]. The levels of serum Cr and blood urea nitrogen (BUN) were increased in KI/R group compared to sham mice (Figure 2F,G); treatment with KYP2047, at both doses of 1 and 5 mg/kg, significantly reduced the high levels of both Cr and BUN in KI/R-injured mice (Figure 2F,G).

### 2.3. The Effects of KYP2047 to Counteract Kidney Fibrosis in AKI

**Figure 3:**
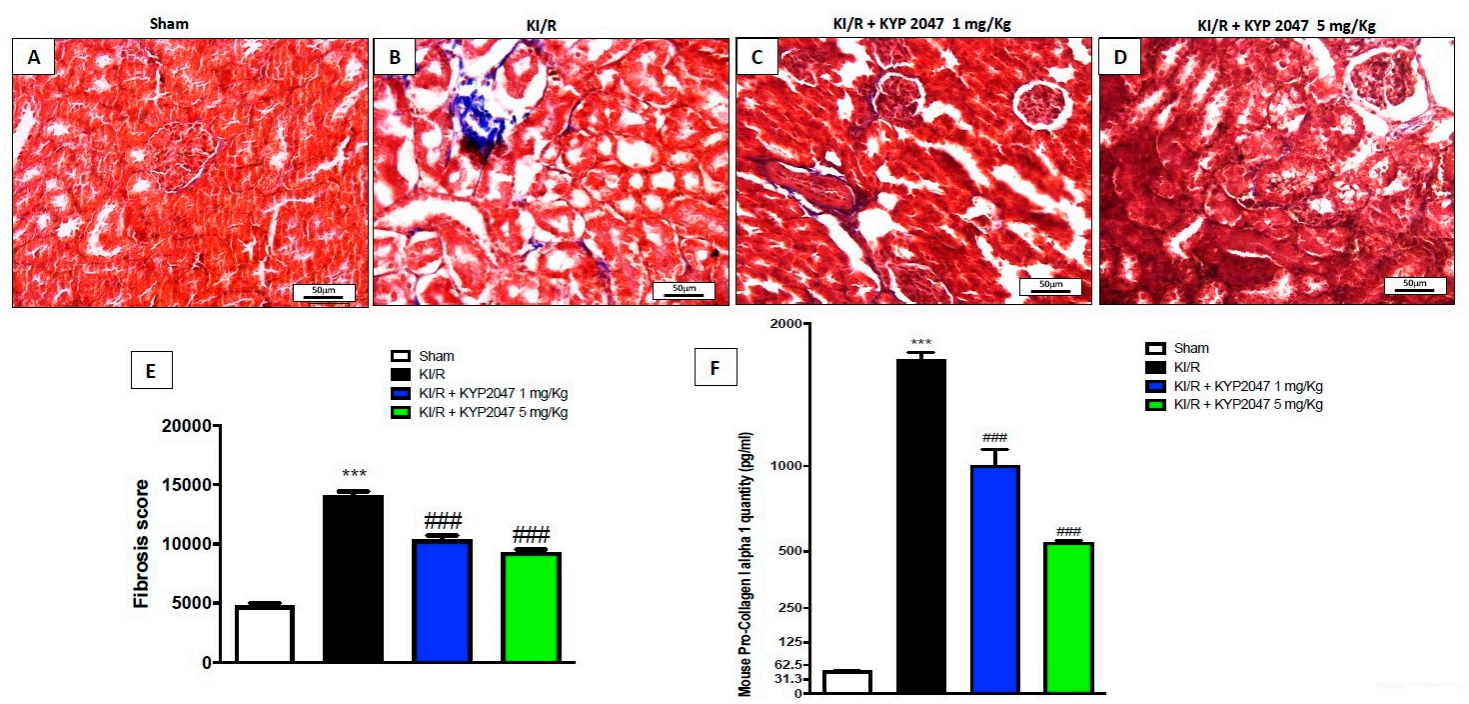
Role of KYP2047 treatment to reduce fibrosis. Masson’s trichrome staining to evaluate fibrotic state following KI/R. Increased collagen deposition was observed in KI/R-injured group (**B**) compared to control group (**A**); reduced collagen content in KYP2047 1 and 5 mg/kg (**C**,**D**); Fibrosis score (**E**). Magnification 20×, scale bar 50 μm (**A**–**D**). Pro-collagen quantity expressed as pg/mL (**F**). Data represent the means of at least three independent experiments. One-way ANOVA followed by Bonferroni post-hoc. *** *p* < 0.001 versus Sham; ### *p* < 0.001 versus KI/R.

KI/R is distinguished by the constantly declining glomerular filtration rate, associated to the transition of postischemic repair into progressive renal fibrosis, characterized by glomerular sclerosis and tubulointerstitial fibrosis [24,25]. Moreover, the upregulation and proliferation of fibroblasts promotes the production and secretion of procollagen I, which cross-links in the extracellular space to form mature collagen, which is a fundamental unit of organ fibrosis [26]. In this study, the fibrosis grade was evaluated through a Masson’s trichrome staining on kidney samples and confirmed by an ELISA kit for the pro-collagen I content, highlighting a notably increase in collagen depot, and in pro-collagen I quantity observed in KI/R injured mice (respectively, Figure 3B, see fibrosis score Figure 3E,F), compared to control animals (respectively, Figure 3A, see fibrosis score Figure 3E,F). The inhibition of POP, mediated by KYP2047 treatment, at both doses of 1 and 5 mg/kg, significantly reduced fibrosis state and the amount of collagen (respectively, Figure 3C,D, see fibrosis score Figure 3E,F).

### 2.4. The Role of POP-Inhibition to Reduce Inflammatory State in KI/R

**Figure 4:**
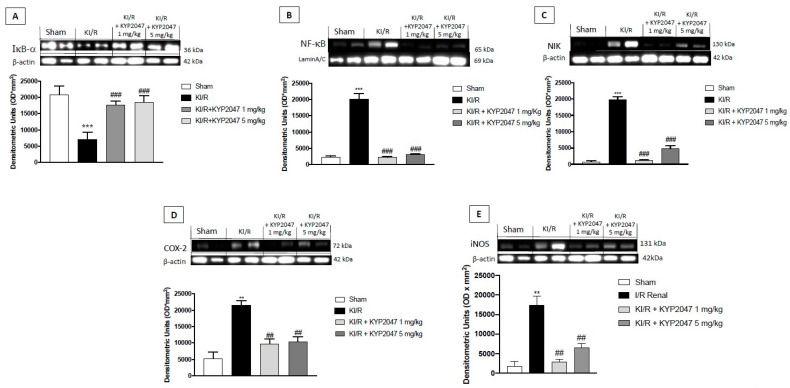
Effects of POP-inhibition on inflammation. Western blot analysis on canonical and non-canonical NF-κB pathways. Reduction of both NF-κB and NIK expressions, compared to KI/R-injured group (**B**,**C**). Contrary, IκB-α cytosolic degradation was observed in KI/R-injured group, significantly prevented by treatment with KYP2047 (**A**). COX-2 and iNOS expressions were upregulated in kidney samples from KI/R-injured mice, compared to control group (**D**,**E**), while treatment with KYP2047 significantly reduced the inflammatory enzyme protein levels (**D**,**E**). Data represent the means of at least three independent experiments. One-way ANOVA followed by Bonferroni post-hoc. *** *p* < 0.001, ** *p* < 0.01 versus Sham; ### *p* < 0.001, ## *p* < 0.01 versus KI/R.

A sterile inflammation occurs frequently in several renal diseases, triggered by ischemia, in response to damage released from injured necrotic cells [27]. POP was also shown to be involved in several physiological and pathological functions, such as inflammation [28]. In kidney injury, inflammatory state is mediated by the crosstalk between canonical and non-canonical NF-κB pathways; the canonical NF-κB pathway always responds rapidly and it is mediated by a kinase complex comprising IKKα, IKKβ, and IKKγ, which phosphorylates the IκB bound to NF-κB dimers, leading to ubiquitination and subsequent proteasome-induced degradation of IκB [29]. Moreover, an important protein, NF-κB-inducing kinase, known as NIK, plays a role in phosphorylation through activation of kinase IKKα [30]. In this study, the anti-inflammatory activity of KYP2047, mediated by POP-inhibition, was observed in the reduction of both NF-κB and NIK expressions, compared to KI/R-injured group (Figure 4B,C). Contrary, the treatment with KYP2047 significantly prevented IκBα cytosolic degradation (Figure 4A), keeping the protein expression at the control levels. Furthermore, research over the years indicates that the major players in the postischemic inflammation are iNOS and COX-2 [31,32]. In this research, the protein expression of COX-2 was significantly upregulated in kidney samples from KI/R-injured mice, compared to control group (Figure 4D), while the treatment with KYP2047 significantly reduced the inflammatory enzyme protein levels at both doses of 1 and 5 mg/kg (Figure 4D). The same result was obtained for the protein expression of iNOS (Figure 4E).

### 2.5. The Effects of KYP2047 Treatment to Modulate Inflammatory Mediators

**Figure 5:**
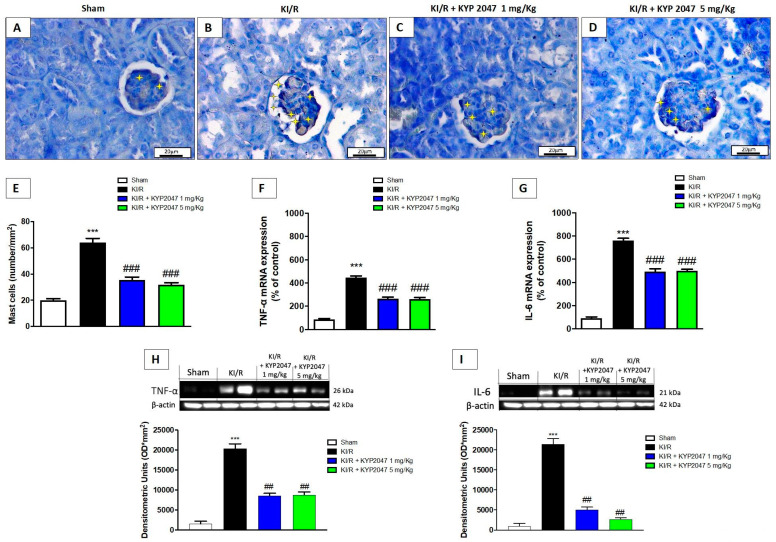
Effects of POP-inhibition on inflammatory mediators. Mast cells degranulation in kidney samples by toluidine blue staining; control (**A**), KI/R-injured group (**B**), KYP2047 at 1 mg/kg (**C**) and 5 mg/kg (**D**). Mast cells graph (number/mm^2^) (**E**). RT-PCR for the evaluation of mast cell–derived TNF-α (**F**) and IL-6 (**G**). Western blot for the evaluation of TNF-α (**H**) and IL-6 (**I**) protein levels. The yellow stars represented the mast cells. Data represent the means of at least three independent experiments. One-way ANOVA followed by Bonferroni post-hoc. *** *p* < 0.001 versus Sham, ## *p* < 0.01 and ### *p* < 0.001 versus KI/R.

The ensuing inflammatory response and a consecutive maladaptive repair and persistent inflammation represents important risk factors for postischemic chronic kidney disease development, characterized by the deleterious role of mast cells [33]. A significant increase of mast cells degranulation was observed in kidney samples from KI/R-injured animals (Figure 5B) compared to control mice (Figure 5A) by toluidine blue staining; the POP-inhibition, mediated by KYP2047, at both doses, significantly reduced mast cell activation (respectively Figure 5C,D). Moreover, mast cell-derived TNF-α results to be a crucial factor in upregulating IL-6, initiating the cytokine cascade responsible for injury [34]. We observed a significant increase in renal TNF-α gene expression in KI/R group compared to control (Figure 5F), while treatment with KYP2047 considerably decreased TNF-α mRNA expression (Figure 5F); the same result was observed for IL-6 mRNA expression (Figure 5G). The changes observed in mRNA pro-inflammatory cytokines reflected the modulation of protein levels; indeed, treatment with KYP2047 significantly decreased TNF-α and Il-6 protein levels compared to KI/R-injured group (respectively, Figure 5H,I).

### 2.6. The Effects of KYP2047 to Modulate Angiogenesis in KI/R

**Figure 6:**
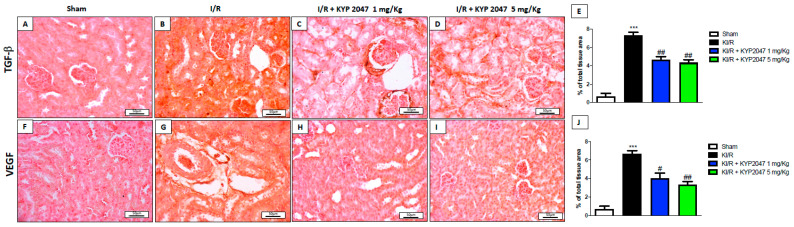
Effects of POP-inhibition on angiogenesis. Immunohistochemical evaluation of TGF-β and VEGF. Increased TGF-β and VEGF positive staining in KI/R-injured group (**B**,**G**) compared to control group (**A**,**F**); reduced TGF-β and VEGF positive staining in treated groups (**C**,**H**,**D**,**I**); % TGF-β total tissue area (**E**) and % VEGF total tissue area (**J**). Magnification 20×, scale bar 50 μm (**A**–**D**,**F**–**I**). Data represent the means of at least three independent experiments. One-way ANOVA followed by Bonferroni post-hoc. *** *p* < 0.001 versus Sham; ## *p* < 0.01 # *p* < 0.05 versus KI/R.

The early stages of IR seem to be associated with an antiangiogenic response, whereas the hypoxia that follows IR at later stages may activate angiogenic factors such as TGF-β and VEGF [11,35]; particularly, angiogenesis has also been implicated in the restoration of ischemic damage and POP has a pro-angiogenic role in this context [36]. In this study, there emerged a positive staining for both TGF-β and VEGF markers (respectively, Figure 6B, see percentage (%) of total tissue area Figure 6E,G, see percentage (%) of total tissue area 6J) compared to control mice (respectively, Figure 6A, see percentage (%) of total tissue area Figure 6E,F, see percentage (%) of total tissue area Figure 6J). The KYP2047 treatment, at both doses of 1 and 5 mg/kg, significantly reduced the positive staining for TGF-β (respectively Figure 6C,D, see percentage (%) of total tissue area Figure 6E) compared to KI/R injured mice. Instead, a slight significant difference in VEGF reduction at the higher treatment dose was observed (Figure 6I, see percentage (%) of total tissue area Figure 6J) compared to the effect promoted by the lowest dose of 1 mg/kg (Figure 6I, see percentage (%) of total tissue area Figure 6J).

### 2.7. The Role of POP-Inhibition to Modulate Apoptosis Related to KI/R

**Figure 7:**
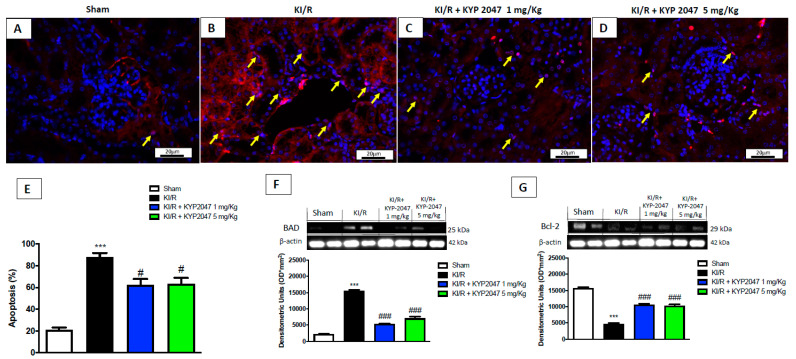
Effects of POP-inhibition on apoptosis. TUNEL staining for apoptosis detection; control (**A**), KI/R-injured group (**B**), KYP2047 at 1 mg/kg (**C**) and 5 mg/kg (**D**). Apoptosis (%) score (**E**). Western blot analysis for Bad (**F**) and Bcl-2 (**G**) and expression (**G**). The yellow arrows represented the cells in apoptosis. Data represent the means of at least three independent experiments. One-way ANOVA followed by Bonferroni post-hoc. *** *p* < 0.001 versus Sham; ### *p* < 0.001 and # *p* < 0.05 versus KI/R.

Apoptosis occurs predominantly as a result of reperfusion, providing additional information regarding the extent of ischemia/reperfusion injury in kidney [37]. The results, obtained through TUNEL staining highlighted an increase in apoptotic cells in samples from he KI/R group (Figure 7B, see graph of percentage (%) apoptosis Figure 7E) compared to control mice (Figure 7A, see graph of percentage (%) apoptosis Figure 7E), while, the treatment with KYP2047, at 1 and 5 mg/kg, notably reduced the apoptotic process, slowing down the damage (respectively, Figure 7C,D, see graph of % apoptosis Figure 7E). This result was confirmed analyzing the protein expression of pro-apoptotic marker Bad (Figure 7F). Meanwhile, Bcl-2 is involved in the mitochondria-mediating intrinsic apoptotic pathway and can inhibit apoptosis in the renal I/R injury [38]; we observed an important role of POP-inhibition to upregulate Bcl-2, so preventing apoptosis process in KI/R, compared to the KI/R-injured group (Figure 7G). 

### 2.8. The Role of POP-Inhibition to Modulate PP2A Activity in KI/R

**Figure 8:**
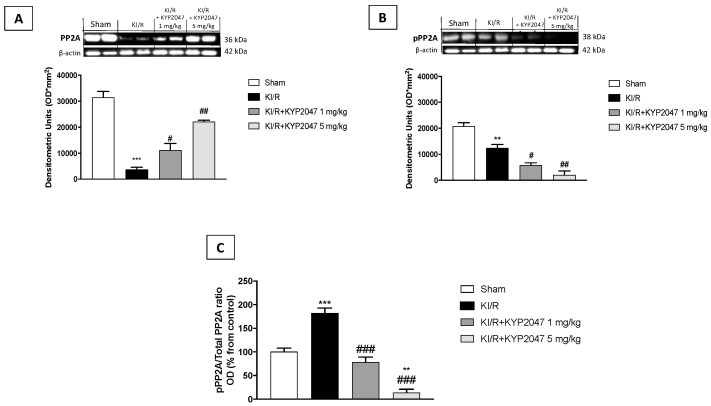
Effects of POP-inhibition on PP2A activity. Western Blot analysis for PP2A detection (**A**), pPP2A detection (**B**), pPP2A/PP2A ratio expressed as percentage of control (**C**). Data represent the means of at least three independent experiments. One-way ANOVA followed by Bonferroni post-hoc. ** *p* < 0.01, *** *p* < 0.001 versus Sham; ### *p* < 0.001, ## *p* < 0.01, and # *p* < 0.05 versus KI/R.

Various studies demonstrated that the physiological role of POP is to regulate PP2A; particularly, POP inhibition was recently shown to increase PP2A activity, thereby reducing oxidative stress [39,40]. Moreover, PP2A serves an important role in protection against renal inflammation [41]. To demonstrate whether PP2A is also activated (i.e., dephosphorylated) by KYP2047 during KI/R, the activated form of PP2A, and the inactivated form (pPP2A) were measured in kidney samples, showing as KYP2047 treatment significantly increased PP2A and reduced pPP2A (respectively, Figure 8A,B). Moreover, the study showed that KYP2047 treatment notably decreased the ratio of phosphorylated PP2A (pPP2A) to total PP2A compared to KI/R-injured group (Figure 8C).

## 3. Discussion

KI/R represents the most common cause of AKI, resulting from a generalized or localized impairment of oxygen, and nutrient delivery to, and waste product removal from, cells of the kidney [42]. The incidence of AKI has grown steadily in many demographic groups and it was estimated to be about 500 per 100,000 individuals [43]. Despite advances in preventive strategies and support measures, AKI continues to be associated with high morbidity and mortality, and in addition to mortality rates, there is a high risk of developing or exacerbating chronic kidney disease [44]. Despite numerous positive research studies regarding the possible therapeutical approach, the translation of this new information into clinical therapies has been slow [45].

AKI, as a dynamic process, involves angiogenesis alteration, inflammatory state activation, endothelial cell injury, and apoptosis, followed by repair that can be adaptive and restore epithelial integrity or maladaptive, leading to chronic kidney disease [46]. Various findings, in different pathological diseases, indicate the involvement of POP in inflammation and vascular modification [16,47,48]. POP has recently gained pharmaceutical interest in different inflammatory models [49,50,51]. Considering that KYP2047 is the most selective inhibitor of POP [47], the aim of this research was to evaluate the protective effect of KYP2047 treatment to counteract inflammation, vascular alterations, and apoptosis process involved in the pathophysiology of AKI through an in vivo mouse model of KI/R.

AKI is considered an inflammatory disease and endothelial cells represents important components of vascular tone and smooth muscle responsiveness in kidney, so that the damage done by KI/R provokes phlogosis, characterized by injury of endothelium, local edema, and arteriolar vasoconstriction [52]. In this study, we confirmed the protective role of KYP2047, at both doses of 1 and 5 mg/kg, to modulate histological alterations due to KI/R. Moreover, it is known that AKI represents an acute renal failure, traditionally described as a rapid decrease in kidney function and measured by increases in serum Cr and urea [53]. This research confirmed a notably alteration in serum Cr and urea levels following KI/R damage, restored by KYP2047 treatment. 

The potential pathologic mechanisms underlying the progression from AKI to CKD include glomerular hyperfiltration and hypertrophy, endothelial injury, and promotion of tubule-interstitial fibrosis [54]. Specifically, deposition of the pathological matrix in the interstitial space and within the walls of glomerular capillaries, as well as the cellular processes resulting in this deposition, are increasingly recognized as important factors amplifying kidney injury and accelerating nephron demise. Moreover, this pathological matrix characterized by deposition of fibrillar substance in the potential space between tubules and peritubular capillaries is rich in fibrillar collagen I [55]. Treatment with KYP2047 significantly reduced fibrosis state and sclerotic lesions, reporting collagen production to a physiological level, highlighting the POP involvement in fibrosis process.

The vascular endothelium plays a central role in the recruitment and migration of circulating inflammatory cells into sites of inflammation [56]. Specifically, inflammation promotes tubulointerstitial fibrosis, which is reflected in reduced number of vessels, associated with chronic hypoxia, and in the compromise of the microvasculature, and due to enhanced tubular stress and epithelial cell injury [57]; therefore, as a repair process, neo-angiogenesis occurs to re-establish the microcirculation that is lost during injury [58]. TGFβ-1-mediated induction of angiogenesis requires a rapid involvement of VEGF and representing a potential target to control angiogenesis [59]. In this study, an increased expression of TGFβ-1 and VEGF was observed in damage conditions, while the treatment with KYP2047 could greatly reduce the cascading events associated with the expression of these angiogenetic mediators. 

TGFβ-1 has long been considered a master cytokine in the pathogenesis of renal inflammation [60]. Generally, ischemic acute renal failure is associated with tubulointerstitial inflammation and studies using animal models have demonstrated that the inflammatory response to I/R exacerbates the resultant renal injury [61]. The inflammatory state in kidney is driven by NF-κB-dependent mechanisms [62]; specifically, NF-κB regulates the expression of numerous genes that play a key role in the inflammatory response during kidney injury and its activity has additionally been linked to AKI, reporting that NF-κB inhibitors reduce AKI severity, even following the start of injury. Multiple stimuli activate NF-κB through the classical pathway in somatic renal cells and noncanonical pathway activation occurs in acute kidney injury [63]. In this study, we confirmed the capacity of KYP2047 treatment to counteract inflammation through a downregulation of canonical NF-κB-pathway, demonstrating, for the first time, an anti-inflammatory capacity of KYP2047 to modulate non-canonical NF-κB-pathway through NIK. Inflammatory enzymes like iNOS and COX-2 is known to mediate the effects of the late phase of ischemia; however, the signaling pathways involved in COX-2 induction following ischemic are unknown. In addition, although iNOS has been identified as a co-mediator together with COX-2, the interaction between iNOS and COX-2 in kidney is unknown. In this study, the anti-inflammatory action of KYP2047 in kidney injury was empowered by a reduction of both inflammatory enzymes.

In the pathophysiology of IRI in kidney, the inflammatory condition is characterized by innate immunity and an adaptive immune response, and implicates mast cells as key regulators. Particularly, it is known that mast cells depletion prior to KI/R resulted in improved renal function due to diminished local inflammatory cytokine/chemokine levels and neutrophil recruitment to the kidneys after the acute injury phase, underlying a deleterious role of mast cells during the acute inflammatory phase of kidney injury [64]. In this study, we proved a significant protective role of POP-inhibition to downregulate mast cells degranulation. This turns out to be very important since mast cells degranulation provokes the release of cytokines and it is known that elevated concentrations of both proinflammatory cytokines IL-6 and TNF-α contribute to the development of Th imbalance and wasting in the uremic milieu [64,65]. For the first time, in this study, we demonstrated that KYP2047 significantly reduced mast cell degranulation and pro-inflammatory cytokines, responsible to strengthen the inflammatory state associated to AKI. 

It is known that PP2A serves an important role in protection against renal inflammation [41] and the developmental regulation of PP2A activity and protein during kidney growth suggests a role for PP2A in the regulation of nephron differentiation [66]. For the first time, in this study, the involvement of PP2A was demonstrated in renal ischemia/reperfusion injury, suggesting an important connection between POP-inhibition and PP2A activity, and strengthening the anti-inflammatory mechanisms of KYP2047 treatment through PP2A activation.

The principal event that leads to inflammatory disease is cell damage and KI/R injury, as a several common renal insult, cause apoptosis in the kidney. Although this programmed cell death is clearly necessary, its dysregulation contributes to atrophy and promotes fibrosis and renal dysfunction; interestingly, proximal tubule epithelial cells are highly susceptible to apoptosis, and injury at this site contributes to organ failure [67]. This research highlighted the capacity of KYP2047, through POP-inhibition, to reduce apoptotic process, by reducing proapoptotic markers and promoting anti-apoptotic mechanisms. 

In conclusion, we can affirm the negative impact of the POP enzyme in the pathogenesis of KI/R and the protective effect of POP-inhibition mediated by KYP2047 treatment in kidney disease, highlighting that POP-inhibition in KI/R involves various mechanisms, inflammation, angiogenesis, and apoptosis, arranging together to better mediate the damage related to kidney I/R. Although, there is evidence for dramatic inactivation of PP2A by KI/R and prevention of this inactivation by POP inhibition, speculating that all the diverse effects of POP inhibition showed in this research may be secondary to the prevention of PP2A inactivation. These data acclaim a considerable anti-inflammatory potential of KYP2047 associated to its modulatory role on angiogenesis and apoptosis in the pathophysiology of AKI, providing new tools in the management of kidney diseases.

## 4. Materials and Methods

### 4.1. Animals 

The study was conducted on adult male CD1 mice (25–30 g, Harlan Nossan, Milan, Italy). The animals were housed in a controlled environment at the laboratories of the University of Messina. Mice were placed in steel cages in a room maintained at 22 ± 1 °C with a 12-h dark and 12-h light cycle and provided with standard rodent food and water. Animal care was approved by the Board of Auditors of the University of Messina and complied with the regulations in Italy (DM 116192), Europe (GU of the EC L 358/1 18/12/1986), and in the United States (Animal Welfare Assurance # A5594-01, United States Department of Health and Human Services).

### 4.2. Kidney Ischemia/Reperfusion (KI/R)

KI/R was performed as previously described [68]. Specifically, mice were anesthetized with a mixture of ketamine (Ketalar, Pfizer, Elsene, Belgium; 80 mg/kg) and xylazine (Rompun, Bayer, Wuppertal, Germany; 16 mg/kg), diluted in sterile saline to a final volume of 2.4 mL/100 g body weight [69], and placed on a heating pad to maintain core body temperature at 37 °C during surgery. The surgery consisted of a midline laparotomy, after which the mice in groups I/R underwent bilateral renal ischemia. Ischemic damage was induced by occluding renal arteries and veins with microaneurysm forceps for 30 min. Timings were chosen based on the literature to maximize the reproducibility of functional renal damage while minimizing mortality in these animals. Five minutes before the reperfusion phase, intraperitoneal treatment was performed using KYP2047 at doses of 0.5, 1, and 5 mg/kg. During reperfusion, the renal clamps were removed, and for the next 5 min, the kidneys were observed to confirm complete reperfusion. Moreover, 1 mL of saline solution at 37 °C was then injected into the abdomen and the incision was sutured in two layers. The mice were allowed to recover under a heat lamp and observed for 6 h.

### 4.3. Experimental Groups

The mice were divided into the following groups:

**Sham**: mice were subjected to midline laparotomy, but not subjected to renal I/R (N = 8);

**KI/R**: mice underwent renal ischemia for 30 min followed by reperfusion for 6 h (N = 10);

**KI/R + KYP2047 (0.5 mg/kg):** mice underwent renal ischemia for 30 min followed by reperfusion for 6 h, and KYP2047 (0.5 mg/kg 0.001% DMSO i.p) was administered five minutes before the reperfusion phase (N = 10);

**KI/R + KYP2047 (1 mg/kg):** mice underwent renal ischemia for 30 min followed by reperfusion for 6 h, and KYP2047 (1 mg/kg 0.001% DMSO i.p) was administered five minutes before the reperfusion phase (N = 10);

**KI/R + KYP2047 (5 mg/kg):** mice underwent renal ischemia for 30 min followed by reperfusion for 6 h, and KYP2047 (5 mg/kg 0.001% DMSO i.p) was administered five minutes before the reperfusion phase (N = 10).

Histological analysis: kidney tissues collected after 6 h of reperfusion were processed for histological analysis, as previously described [47]. Briefly, samples were fixed in formaldehyde buffered solution (10% phosphate buffered saline) at room temperature, dehydrated with graded ethanol, and included in paraffin. Subsequently tissue sections (7 μm thick) were deparaffinized with xylene, stained with hematoxylin/eosin, and studied with light microscope (Zeiss Milan, Italy). For the quantitative estimation of I/R lesions, histological studies were performed by two independent observers blinded to the experimental protocol. The morphological criteria were used as previously described [70]. Briefly, 100 intersections were examined and a score of 0 to 3 was assigned for each tubular profile involving an intersection: 0, normal histology; 1, tubular cell swelling, brush edge loss, nuclear condensation, with up to one-third of the tubular profile showing nuclear loss; 2, as with score 1, but greater than one-third and less than two-thirds of the tubular profile showing nuclear loss; 3, more than two-thirds of the tubular profile showing nuclear leaks. The total score for each kidney was calculated by adding all 100 scores with a maximum score of 300.

### 4.4. PAS Staining

PAS staining allows highlighting the glycogen deposits, providing information on the general structure of the tissues. Renal sections were scored by three blinded individuals ranging from 0, no staining of glycogen granules, to 3, the most intense staining. For PAS staining, 20× magnification (50 µm scale bar) is shown. All analyses were performed by two observers, who were blinded to the treatment [71].

### 4.5. Assessment of Renal Function 

Serum creatinine and blood urea nitrogen levels (BUN) were estimated from blood samples as previous described [72].

### 4.6. Blue Toluidine Staining

Renal tissues were stained with toluidine blue (Bio-Optica, Milan, Italy) to assess the quantity of mast cells and their degranulation, as previously described [73]. A base dye colors sections blue and highlights purple mast cells. Renal tissues were deparaffinized in xylene and dehydrated using ethanol graded sequences. They were then placed in water for 5 min and stained with toluidine blue for 4 min. Sections were placed in absolute alcohol for 1 min, clarified in xylene, and fixed on slides using Eukitt (Bio-Optica, Milan, Italy). The number of metachromatic colored mast cells was obtained by counting five high-power fields per section, using an AxioVision Zeiss microscope (Milan, Italy) and the related AxioVision software (Carl Zeiss Vision, Jena, Germany). Data were reported as mean with standard deviation (SD). For toluidine blue staining, the results were shown at 40× magnification (20 µm scale bar).

### 4.7. Masson’s Trichrome Staining

The degree of fibrosis of the renal sections was assessed using Masson’s trichrome stain, following the manufacturer’s instructions (Bio-Optica, Milan, Italy), as previously described [73,74]. This staining allows highlighting connective tissue, collagen, reticular fibers, and muscle fibers. The results are shown at 20× magnification (50 µm scale bar).

### 4.8. ELISA Kit for Pro-Collagen 1 Evaluation

Mouse Pro-Collagen I alpha 1 ELISA Kit (Abcam, #ab210579) was used to evaluate the collagen content in kidney samples, as previous described [75]. In detail, samples were thawed on ice and homogenized in 300 μL lysis buffer (750 μL, Pierce #87787, Thermo Fisher Scientific, Waltham, MA, USA) supplemented with a protease inhibitor cocktail (Sigma-Aldrich, Rehovot, Israel). Thereafter, kidney samples were homogenized and centrifuged at 14,000× *g* for 10 min at 4 °C; supernatants were collected, aliquoted, and subjected to analysis.

### 4.9. Western Blot Analysis

The cytosolic and nuclear extracts of kidney tissues collected for western blot analysis were obtained as previously described [76,77]. The expression of nuclear factor of kappa light polypeptide gene enhancer in B cell inhibitor alpha (IκB-α), inducible nitric oxide synthase (iNOS), cyclooxygenase 2 (COX-2), NF-κB-inducing kinase (NIK), B-cell lymphoma 2 (Bcl-2), Bcl-2 associated agonist of cell death (BAD), tumor necrosis factor (TNF-α), interleukine-6 (IL-6), protein phosphatase 2 (PP2A), and p-PP2A were quantified in cytosolic fraction. Membranes were then incubated with peroxidase-conjugated bovine anti-mouse secondary antibody or peroxidase-conjugated goat anti-rabbit IgG (1:2000, Jackson ImmunoResearch, Jackson Laboratories Bar Harbor, ME, Bar Harbor, USA) for 1 h at room temperature [78]. Nuclear factor kappa-light-chain-enhancer of activated B cells (NF-κB) was quantified in nuclear fraction. The filters were probed with specific Abs: anti-NF-κB (1:500; Santa Cruz Biotechnology sc-8008) or IκB-α (1:500; Santa Cruz Biotechnology sc-1643), anti-COX-2 (1:500; Cayman 160106) or iNOS (1:500, Abcam ab3523), anti-Bcl-2 polyclonal antibody (1:500 Santa Cruz Biotechnology sc-7382), anti-Bad (1:500, Santa Cruz Biotechnology sc-8044), anti TNF-α (1:500, Santa Cruz Biotechnology sc-52746), anti-IL-6 (1:500, Santa Cruz Biotechnology sc-57315) PP2A (1:500; Merck 05–421), and p-PP2A (1:500; Thermo Fisher Scientific PA5-36874) in 1 × PBS, 5% *w/v* non-fat dried milk, 0.1% Tween-20 at 4 °C, overnight. To ascertain, those blots were loaded with equal amounts of proteins, they were also incubated in the presence of the antibody against GAPDH (cytosolic fraction 1:500; Santa Cruz Biotechnology sc-47724) or lamin A/C (nuclear fraction 1:500 Santa Cruz Biotechnology sc-376248.).

### 4.10. TUNEL Staining 

The TUNEL staining protocol was performed following the manufacturer’s instructions (Roche), as previously described [79]. The sections were deparaffinized in xylene and serially hydrated with ethanol in water, permeabilized with 0.1 M citrate buffer, and incubated in the TUNEL reaction for 60 min at 37 °C, in the dark. The samples were rinsed in PBS and then observed using an excitation wavelength in the range 520–560 nm (maximum 540; green) and in the range 570–620 nm (maximum 580 nm; red). For TUNEL staining, 40× magnification (20 µm scale bar) is shown. All analyses were performed by two observers, who were blinded to the treatment.

### 4.11. Immunohistochemical Localization of TGF-β and VEGF

Immunohistochemical localizations of transforming growth factor beta (TGF-beta) and vascular endothelial growth factor (VEGF) were determined, as previously described [80]. Sections (7 μm) were prepared from paraffin-embedded tissues. After deparaffinization, endogenous peroxidase was quenched with 0.3% hydrogen peroxide in 60% methanol for 30 min. The sections were permeabilized with 0.1% Triton X-100 in PBS for 20 min. Sections were incubated overnight with anti-TGFβ (Santa Cruz Biotechnology, 1:100 in PBS, *v/v*) and anti-VEGF (1:100, Santa Cruz Biotechnology, in PBS, *v/v*). Sections were washed with PBS, and incubated with secondary antibody. Specific labeling was detected with a biotin-conjugated goat anti-rabbit IgG and avidin-biotin peroxidase complex (Vector Lab Inc., Burlingame, CA, USA). Images were collected using a Zeiss microscope and Axio Vision software. For graphic display of densitometric analyzes, the percentage (%) of positive staining (brown staining) was measured by computer-assisted color image analysis (Leica QWin V3, UK). The percentage area of immunoreactivity (determined by the number of positive pixels) was expressed as a percentage (%) of the total tissue area (red staining) within five random fields at 20× magnification. In particular, firstly, the colors of the images that were stained to the molecule of interest were defined. Once these colors were defined, they were automatically detected in all samples. 

Total RNA of kidney was isolated according to the manufacturer’s instructions (RNeasy Mini Kit; Qiagen). RNA concentration was determined using the NanoDrop^TM^ 1000 (Thermo Scientific, Waltham, MA, USA). Four micrograms of total RNA was reverse-transcribed into cDNA, according to the manufacturer’s instructions (PrimeScript RT Master Mix, Takara, Japan).

### 4.12. Real-Time PCR

Real-time PCR amplifications were conducted with the use of the ABI 7500 system (Applied Biosystems, Waltham, MA, USA), as previous described [81]. Briefly, the reaction mixture contained 4 µL of diluted cDNA, 5 pm of each primer, and 10 µL of 2X SYBR green master mixes in a total volume of 20 µL. PCR was conducted at 95 °C for 15 min, followed by 40 cycles at 95 °C for 15 s, and 58 °C for 1 min. This program was followed by analysis of the melting curve that was performed with linear heating from 60 to 90 °C. This analysis was performed to measure TNF-α and IL-6 mRNA expressions in the kidney samples. PCR primers for all analyzed genes were:

TNF-α gene: GTGATCGGTCCCAACAAGGATGGTGGTTTGCTACGACGTG

IL-6 gene: AAGTCCGGAGAGGAGACTTCAGCCATTGCACAACTCTTTTCTCATT

### 4.13. Statistical Analysis

The values shown in the figures and in the text are expressed as the mean–standard error of the mean (SEM) of N observations. For in vivo studies, N represents the number of animals studied. The values shown in the experiments of histology or immunohistochemistry are representative of at least three experiments performed on different experimental days. Results were analyzed by one-way ANOVA followed by a Bonferroni post-hoc test for multiple comparisons. Non-parametric data will be analyzed with Fisher’s exact test. A *p* value of less than 0.05 will be considered significant.

## Data Availability

The data presented in this study are available on request from the corresponding author. The data are not publicly available.
